# Genomic signatures of hybridization between *Ixodes ricinus* and *Ixodes persulcatus in natural populations*

**DOI:** 10.1002/ece3.11415

**Published:** 2024-05-20

**Authors:** Theophilus Yaw Alale, Jani J. Sormunen, Eero J. Vesterinen, Tero Klemola, K. Emily Knott, Miguel Baltazar‐Soares

**Affiliations:** ^1^ Department of Biology University of Turku Turku Finland; ^2^ Biodiversity Unit University of Turku Turku Finland; ^3^ Department of Biological and Environmental Science University of Jyväskylä Jyväskylä Finland

**Keywords:** ddRAD, genetic admixture, hybridization, *Ixodes persulcatus*, *Ixodes ricinus*, polymorphisms

## Abstract

Identifying hybridization between common pathogen vectors is essential due to the major public health implications through risks associated with hybrid's enhanced pathogen transmission potential. The hard‐ticks *Ixodes ricinus* and *Ixodes persulcatus* are the two most common vectors of tick‐borne pathogens that affect human and animal health in Europe. *Ixodes ricinus* is a known native species in Finland with a well‐known distribution, whereas *I. persulcatus* has expanded in range and abundance over the past 60 years, and currently it appears the most common tick species in certain areas in Finland. Here we used double‐digest restriction site‐associated DNA (ddRAD) sequencing on 186 ticks (morphologically identified as 92 *I.* ricinus, and 94 *I. persulcatus*) collected across Finland to investigate whether RAD generated single nucleotide polymorphisms (SNPs) can discriminate tick species and identify potential hybridization events. Two different clustering methods were used to assign specific species based on how they clustered and identified hybrids among them. We were able to discriminate between the two tick species and identified 11 putative hybrids with admixed genomic proportions ranging from approximately 24 to 76 percent. Four of these hybrids were morphologically identified as *I. ricinus* while the remaining seven were identified as *I. persulcatus.* Our results thus indicate that RAD SNPs are robust in identifying both species of the ticks as well as putative hybrids. These results further suggest ongoing hybridization between *I. ricinus* and *I. persulcatus* in their natural populations in Finland. The unique ability of RAD markers to discriminate between tick species and hybrids adds a useful aspect to tick evolutionary studies. Our findings align with previous studies and suggest a shared evolutionary history between the species, with instances of individuals possessing a considerable proportion of the other species' genome. This study is a significant step in understanding the formation of hybridization zones due to range expansion potentially associated with climate change.

## Graphical abstract

Co‐occurrence of the two hard tick species (*Ixodes ricinus* and *Ixodes persulcatus*) increases hybridization potential in natural populations. Species range expansion may drive genomic hybridization between *Ixodes ricinus* and *Ixodes persulcatus* in natural populations.

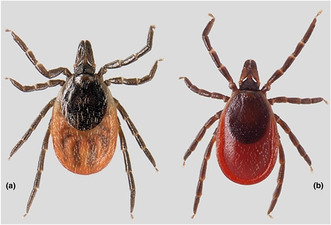

## 1 INTRODUCTION

Hybridization involves the exchange of genetic material between closely related species, leading to the production of admixed genomes potentially impacting genome evolution (Mixão & Gabaldón, 2018; Runemark et al., 2019). While in some cases the impacts of hybridization events can be considered inconsequential from the human perspective, in others they can pose a threat for native flora and fauna, calling for human intervention and preventive measures, such as in cases where rare native or endemic species are threatened by extinction through hybridization (Rhymer & Simberloff, 1996). Of particular interest are cases of hybridization between vectors of zoonotic diseases since they can lead to pathogen spillover or changes in the behaviour of the vectors (Borlase et al., 2021). In this sense, hybrid identification among disease vectors is essential due to the major public health risks associated with them. For example, it has been observed that hybrid individuals within *Culex pipiens* complex mosquito populations possessed an enhanced transmission of the West Nile Virus compared to one or both parental strains (Ciota et al., 2013). Hybridization can have significant impact on host species diversification (Arnold, 2004), affect speciation and the emergence of unique pathogen species (Stukenbrock et al., 2012), and influence disease pathogen transmission (Ciota et al., 2013). As a source of novel genetic variation, hybridization often serves as the basis for natural selection to determine the evolution of important traits (Arnold, 2007; King et al., 2015). Specifically, hybridization events have been shown to introduce beneficial alleles directly into species populations due to new allelic combinations (King et al., 2015); to enhance geographic range expansions in spider populations (Krehenwinkel & Tautz, 2013); promote adaptations to new environments as in the case of Darwin finches (Grant & Grant, 2008).

**FIGURE 1:**
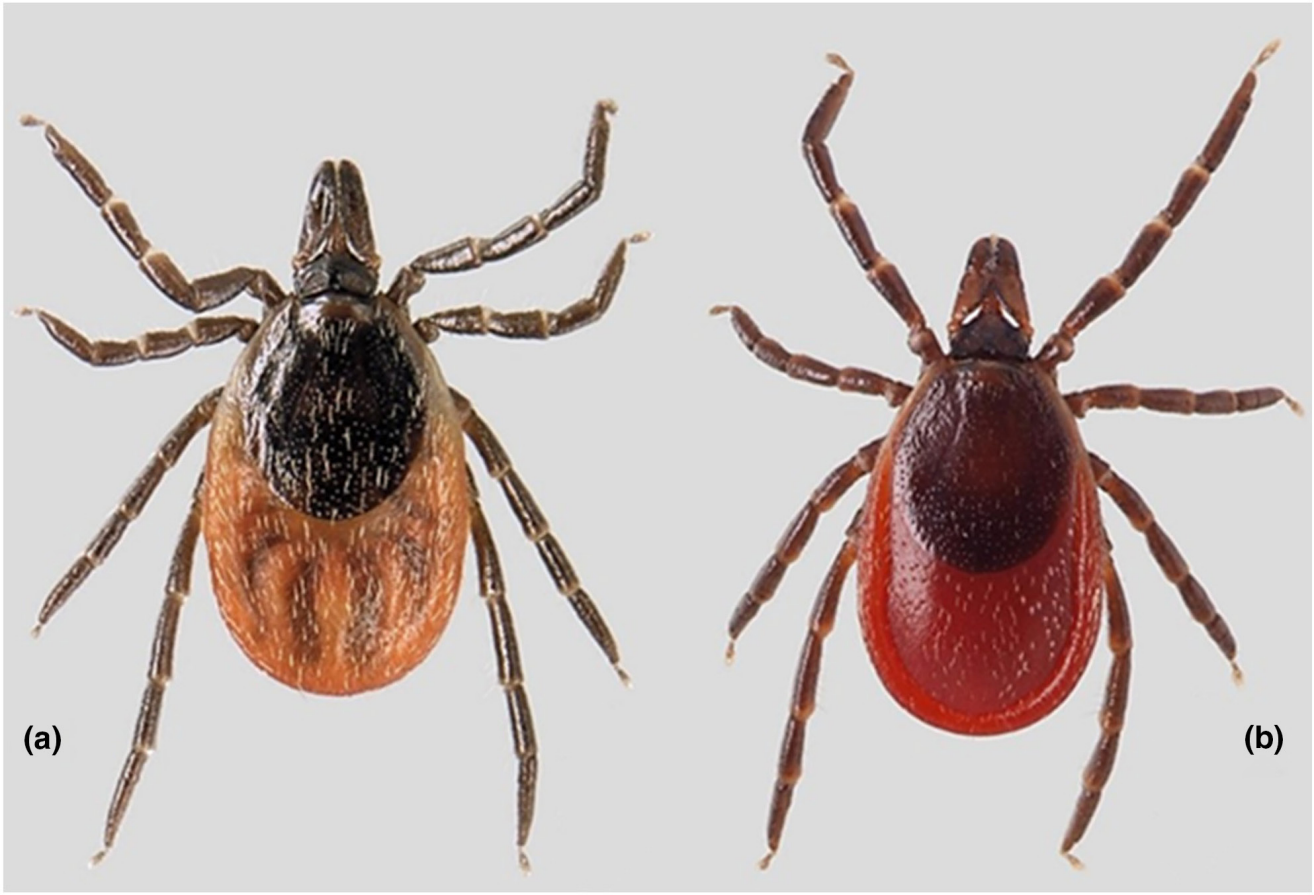
An image of the two *Ixodes* tick species used in this study. *I. ricinus* adult female (a); *I. persulcatus* adult female (b). Image provided by the university of Turku ticks and tick‐borne pathogens research group.

Hard ticks (Acari: Ixodidae) are essential arthropod vectors of medically important pathogens due to their ability to host several pathogenic viruses, bacteria, and protozoa (Araya‐Anchetta et al., 2015). The most notable tick‐borne pathogens in Europe include the pathogenic *Borrelia burgdorferi* sensu lato spirochetes causing Lyme borreliosis and the tick‐borne encephalitis virus (TBEV) causing tick‐borne encephalitis (TBE). Multiple pathogens in an individual tick can result in the transmission of multiple pathogens to humans or other animals after just one bite. In Finland, the castor bean tick *Ixodes ricinus* (Linnaeus, 1758) (Figure 1a) and taiga tick *Ixodes persulcatus* (Schulze,1930) (Figure 1b) are the two main vectors for tick‐borne pathogens and their geographical distributions are well known (Kulha et al., 2022; Laaksonen et al., 2018; Sormunen et al., 2020). Overlapping geographical ranges of these tick species create sympatric zones, which might lead to increased sharing of pathogens or pathogen strains between the tick species (when feeding on the same hosts), increased pathogen loads within each tick species (when ticks are exposed to more hosts carrying pathogens) and to higher genetic diversity of the pathogens due to increased population sizes (when there are more ticks and host species to infest) (Ogden et al., 2013).

As generalist species (Mihalca et al., 2017), *I. ricinus* and *I. persulcatus* are known to parasitize a wide variety of host animals and share a common ecological niche (Laaksonen et al., 2017, 2018; Uspensky, 1993). The two species also share similar morphological features, but also reproductive compatibility leading to interspecific crossing and hybridization (Balashov et al., 1998; Bugmyrin et al., 2013). Within the genus *Ixodes*, Kovalev et al. (2015) identified interspecific hybridization between natural populations of *I. persulcatus* and *I. ricinus* in Estonia, while Patterson et al. (2017) showed evidence of hybridization between *I. scapularis* and *I. cookei* in Canada. *Ixodes ricinus* is a native tick species in Finland and in most parts of Europe, while *I. persulcatus*, which is originally a native species in Asia, has established populations in Finland and in other parts of Europe over the past decades (Kovalev et al., 2015). In Finland, the first nationwide study on the distribution of ticks was carried out in the late 1950s (Öhman, 1961). While this was a questionnaire‐based study, some field studies were conducted in Eastern Finland. Only *I. ricinus* was identified during these studies. However, a crowdsourcing‐based study conducted in 2015 identified *I. persulcatus* from many areas in Finland, with the species appearing to even be dominant in certain areas (Laaksonen et al., 2017). Interestingly, for *I. persulcatus*, the distribution of the species seems to have expanded westwards over Finland during the past 50–60 years, with the first reports of established populations in Sweden being published in 2016 (Jaenson et al., 2016). The establishment of *I. persulcatus* in Finland has naturally led to increased probability of hybridization between the species. Interspecific hybridization between the two species could impact vector competence by introducing novel alleles, widen host use due to increased aggression, or affect the ability to exploit diverse microhabitats (Kovalev et al., 2016). Although this has not been tested, the quantum evolutionary hypothesis of TBEV perceives tick hybrids as a bridge vector required for the emergence of European subtypes of TBEV (Kovalev & Mukhacheva, 2014).

Population genetics of vectors provide useful tools for understanding species differentiation and host–parasite evolution (Gloria‐Soria et al., 2022; Jia et al., 2020; Poli et al., 2020; Zhang et al., 2022). In studies of tick vectors, much attention has been given to identifying levels of genetic differentiations, and how genetic variations affect tick species dispersal and distribution within a specified geographic range (Araya‐Anchetta et al., 2015). Most of these studies have mainly relied on using mitochondrial genetic markers. For tick species identification, different mitochondrial and rRNA‐based markers have been used including COI, 16SrDNA, ITS2, and 12SrDNA (Kovalev et al., 2015; Litov et al., 2022; Lv et al., 2014; Patterson et al., 2017). While mtDNA and rDNA markers have been extensively used for tick species identification, they are somewhat limited in utility due their maternal inheritance and the likely non‐recombining nature of mtDNA, as well as potential linkage to sex chromosomes reducing efficiency regarding male hybrid identification (Hurst & Jiggins, 2005; Litov et al., 2022; Rubinoff et al., 2006).

Restriction site associated DNA sequencing (RADseq) (Baird et al., 2008) is a reduced‐representation technique which relies on a high throughput sequencing approach to generate genome‐wide genetic data that is widely used in the field of population genetics (e.g., Andrews et al., 2016; Narum et al., 2013). RADseq has been used for hybridization detection in many cases, including, to investigate the levels of interspecific gene flow in an endemic cichlid fish (Ford et al., 2015)., investigate interspecific hybridization and phylogeny in the cryptic land snail genus *Pyramidula* (Razkin et al., 2016); and, infer phylogeny and introgression in flowering plants (*Pedicularis*: Orobanchaceae) (Eaton & Ree, 2013); and hybridizations between two Australian reptile ticks, *Amblyomma albolimbatum* and *A. limbatum* (Barnden et al., 2023). Importantly, findings from Barnden et al. (2023) indicated that RADseq‐derived SNPs successfully identified hybrids and separated the two species. Although the method has a wide applicability and the utility of RAD generated SNPs has been shown in several studies, their use in the study of tick hybridization is still limited.

In this study, we used double‐digest RADseq (ddRADseq) (Peterson et al., 2012) to investigate whether (1) RAD generated SNPs are sufficient for identifying the two tick species, *I. ricinus* and *I. persulcatus* and whether (2) RAD generated SNPs are robust for identifying hybrids among the sampled individuals. We hypothesize that the RAD generated SNPs are sufficient to assign the two tick species, their hybrids and identify genetic structure between species.

## 2 MATERIALS AND METHODS

### 2.1 Sampling strategy

**FIGURE 2:**
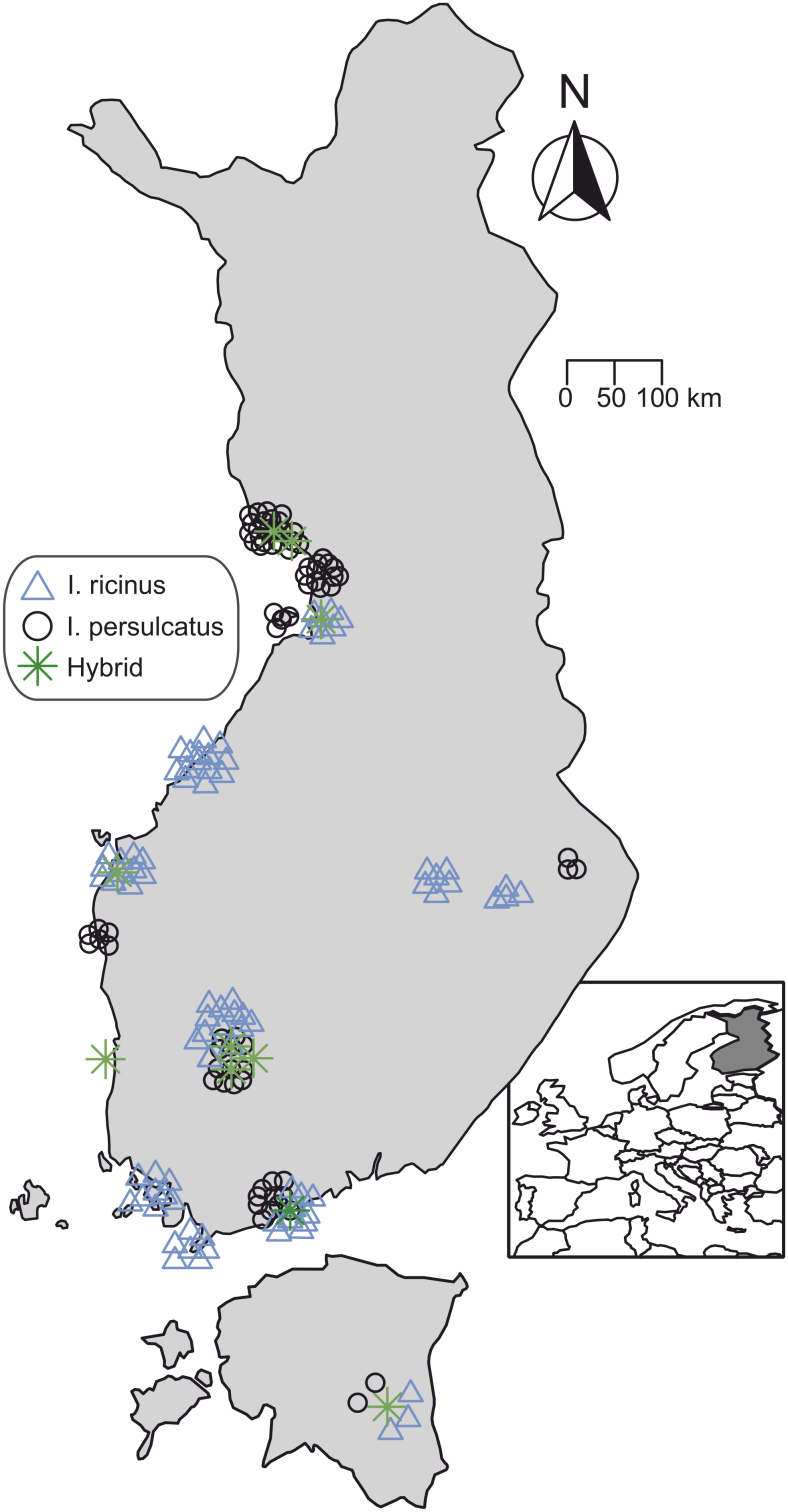
Geographical distribution of Finnish samples. Colour codes indicate species assigned to these locations.

Sample collection largely follows Laaksonen et al. (2017). Briefly, as part of a citizen science campaign led by the University of Turku Tick Project in 2015 (https://sites.utu.fi/puutiaiset/en), ticks were sent to the zoological museum of University of Turku, Finland, by post from different geographical zones and regions across Finland. All samples were morphologically identified to species level (Laaksonen et al., 2017), whiles morphologically unrecognized samples were identified using qPCR (Laaksonen et al., 2018). Upon arrival and species identification at University of Turku, the tick samples were stored at −80°C prior to DNA and RNA extractions. A geographically representative sub‐sample from the citizen science campaign was chosen for this study, based on the available information of tick distribution in Finland (Laaksonen et al., 2017). In addition to samples from the citizen science campaign (*n* = 172), some *I. ricinus* (*n* = 6) and *I. persulcatus* samples (*n* = 8) were received from a collaborator in Estonia and included in the analyses. In total, analyses included 186 samples, comprising 92 *I. ricinus* and 94 *I. persulcatus*. Finnish samples were collected from different hosts, including dogs, cats and humans, while Estonian samples were from unknown hosts or field collected (Table S1). The final sample composition for analyses, after quality checking (see below), included 92 *I. ricinus* (78 adult females, 11 adult males, 3 nymphs), and 94 *I. persulcatus* (79 females, 13 males and 2 nymphs). The geographical origin of samples used in this study are shown in Table S1. The geographical locations of samples with potential hybrid zones have been shown in Figure 2.

### 2.2 DNA extraction, library preparation and RADtag sequencing

Total DNA was extracted from the tick samples using NucleoSpin RNA kits and DNA buffer sets (Macherey‐Nagel, Düren, Germany), following the kit protocols (RNA Kit: Rev. 16 May 2014; DNA buffer set: Rev. 09 April 2017). DNA extracts were stored at −20°C.

Genomic DNA was prepared for genotyping‐by‐sequencing using a double digestion RAD‐seq method adapted from Elshire et al. (2011) and Lemopoulos et al. (2018). These methods support the use of low‐concentration samples. For the digestion step, 90 ng of tick sample was used in a reaction together with 20 U of each of the restriction enzymes PstI and BamHI (New England Biolabs). In the ligation step, the total volume was reduced to 30 μL by vacuum centrifugation, consisting of 12 μL digested DNA, 1× T4 ligation buffer, 720 U of T4 ligase and 0.5 pmol of both forward and reverse adapters. In the pooling step 96 uniquely barcoded samples were pooled by combining 10 μL of each sample, and the pooled sample was purified using four columns of Qiagen QIAquick PCR purification kit, eluting each column in 40 μL. The pool was then PCR‐amplified using tailed primers for adding on Illumina adapters. The 25 μL amplification reaction consisted of 0.5 U Phusion® High‐Fidelity DNA Polymerase with 1× Phusion HF buffer (Thermo Fisher Scientific), 0.2 mM dNTP each and 3 μL of template. The PCR profile included an initial step for nick translation at 72°C for 5 min, denaturation at 95°C for 30 s, followed by 15 cycles of 98°C for 10 s, 64°C for 10 s and 72°C for 5 s. The amplified library was then selected with the Pippin Prep system using CDF 2010 Gel Cassettes (Sage Science), targeting 250–450 bp fragments. PstI‐BamHI‐digested libraries were prepared by the Center of Evolutionary Applications (University of Turku) and sequenced using the Illumina Hiseq2500 platform. Libraries were sequenced over two lanes (one pool of 96 samples per each lane) with 100 bp single end reads at the Finnish Functional Genomics Center (Turku Bioscience).

### 2.3 Bioinformatic processing and filtering

Initial bioinformatic processing of raw reads, such as de‐multiplexing of individual barcodes, adaptors, and barcode trimming, was done at the Finnish Functional Genomics Center (Turku Bioscience). All bioinformatic data processing was done on the CSC supercomputing platform (CSC.fi). Downstream RAD sequence data processing was done using the *Stacks* software package version 2.60 (Catchen et al., 2013). We followed the stacks pipeline for quality filtering and removal of ambiguous samples (Catchen et al., 2013). For reference‐based alignments, reference genomes with the Genbank accession numbers PRJNA270959 (Cramaro et al., 2015) and PRJNA633311 (Jia et al., 2020) for *I. ricinus* and *I. persulcatus* respectively, were downloaded from NCBI's Genbank. RADseq reads were first aligned against each species reference genome using BWA‐mem 2/2.2 algorithm (Li, 2013). The *I. persulcatus* reference genome was eventually chosen for downstream analysis based on the alignment quality scores. To ensure that our results are not biased towards *I. persulcatus*, we further used the *I. scapularis* reference genome (accession number: PRJNA678334) (De et al., 2023) to analyse all the samples and the results were similar for both species assignment and putative hybrids detection (see supplementary material section B). The *Stacks* version 2.60 (Catchen et al., 2013) pipeline was used for variant calling by specifying the following filtering parameters: –min–maf = 0.05, *R* = .8, *p* = 1, – *max*‐obs‐het = 0.5 and retaining only one random SNP per loci (Rochette & Catchen, 2017). Low quality reads after mapping to reference genomes (MAPQ < 20) were excluded from downstream analysis. Due to the high number of repeats in the tick genome (Cramaro et al., 2015; De et al., 2023; Gulia‐Nuss et al., 2016; Jia et al., 2020), all reads which had coverage above 100× were removed.

### 2.4 Clustering analysis and genetic structure

To identify the two species and their putative hybrids based on RADseq markers, we used two separate clustering methods. We first used Discriminant Analysis of Principal Components (DAPC) (Jombart et al., 2010) with the R package ‘*adegenet*’ (Jombart & Ahmed, 2011) in R (R Core Team, 2022). Based on the BIC values obtained (Figure S1) with the find.cluster function (Jombart et al., 2010), the optimal *K* clusters were identified from the *K*‐means. Maximum assumed clusters were allowed based on all sampling locations. Further, we used the *compoplot* function in ‘*adegenet’* to investigate group assignments based on their membership probabilities to have a general overview of cluster composition (Figure S2).

We also employed a nonspatial Bayesian clustering algorithm implemented in the computer program *STRUCTURE* version 2.3.4 (Pritchard et al., 2000). This model uses a Markov chain Monte Carlo to infer genetic clusters and assign individuals to ancestral populations while minimizing Hardy–Weinberg and linkage disequilibrium within clusters (Pritchard et al., 2000). We selected the admixture model and assumed allele frequencies were correlated (Falush et al., 2003). We performed 100,000 Markov chain Monte Carlo burn‐in repetitions to reduce initial configuration effects, followed by 100,000 Markov chain Monte Carlo replications. Based on the number of principal components (PCs) initially obtained, we set *K* from 1 to 6 with five iterations for each *K*. We calculated the arithmetic mean and standard deviation of the log probability of the data (Ln P(D)) across runs for each *K* to identify the plateau and determine the optimal number of clusters (Pritchard et al., 2000). We also calculated the Delta *K* statistic (Evanno et al., 2005) and used ancestry proportions (i.e. *q*‐values) as an index (Pritchard et al., 2000) to inform the selection of *K*. To identify the most likely number of clusters (*K*), we used the online tool, Structure Harvester (Earl & vonHoldt, 2012) to analyse the structure output result (Figure S3). We identified the optimal clusters from the best *k* value by comparing the log probabilities from the results of each *K* value and Evano's delta *K* (ln [Pr(X|*K*)]) approach (Evanno et al., 2005). Following the suggestion of Pritchard et al. (2000), we aimed at the least value of *K* that represents most of the genetic groupings from the data to choose the best *K* clusters (Table S2).

### 2.5 Population genetic diversity

We used the Arlequin population genetics software v3.5.2.2 (Excoffier & Lischer, 2010) to measure within species pairwise Wright's *F*‐statistics (*F*_ST_) (Weir & Cockerham, 1984). *F*_ST_ estimates were computed with 10,000 bootstrapping permutations with statistical significance level *p* = .01. This was done for pure species by removing all putative hybrids from the samples (*n* = 175 comprising of 88 *I. ricinus* and 87 *I. persulcatus*). We also estimated genetic diversity indices from the *Stack*s' *populations* program. For each locus we estimated the observed heterozygosity (*Ho*), number of private alleles (*pA*) and the number of variant sites (vS). Heterozygosity here means individual level genetic diversity, private alleles are alleles found only in one of many populations (Neel, 1973), and variant sites refer to specific regions of the genome which differs between two genomes.

## 3 RESULTS

### 3.1 Sequencing quality and SNPs calling

A total of 186 ticks of both species were kept for all bioinformatic analysis after alignment and quality filtering. The sequencing run produced a total of 292.8 million de‐multiplexed single‐end reads. On average, 1.6 M reads (SD ± 0.83) per individual mapped sequences were obtained. Only 108 M representing a percentage rate of 37.2% of the total aligned reads were kept after the filtering step. The overall per‐sample mean coverage was found to be 51.4× (SD = 29.5×). These reads were initially genotyped into a total of 240,695 loci, of which 3210 loci remained after quality filtering, subsequently resulting in 9760 quality SNPs for downstream analysis.

### 3.2 Species assignment and hybrid identification

**FIGURE 3:**
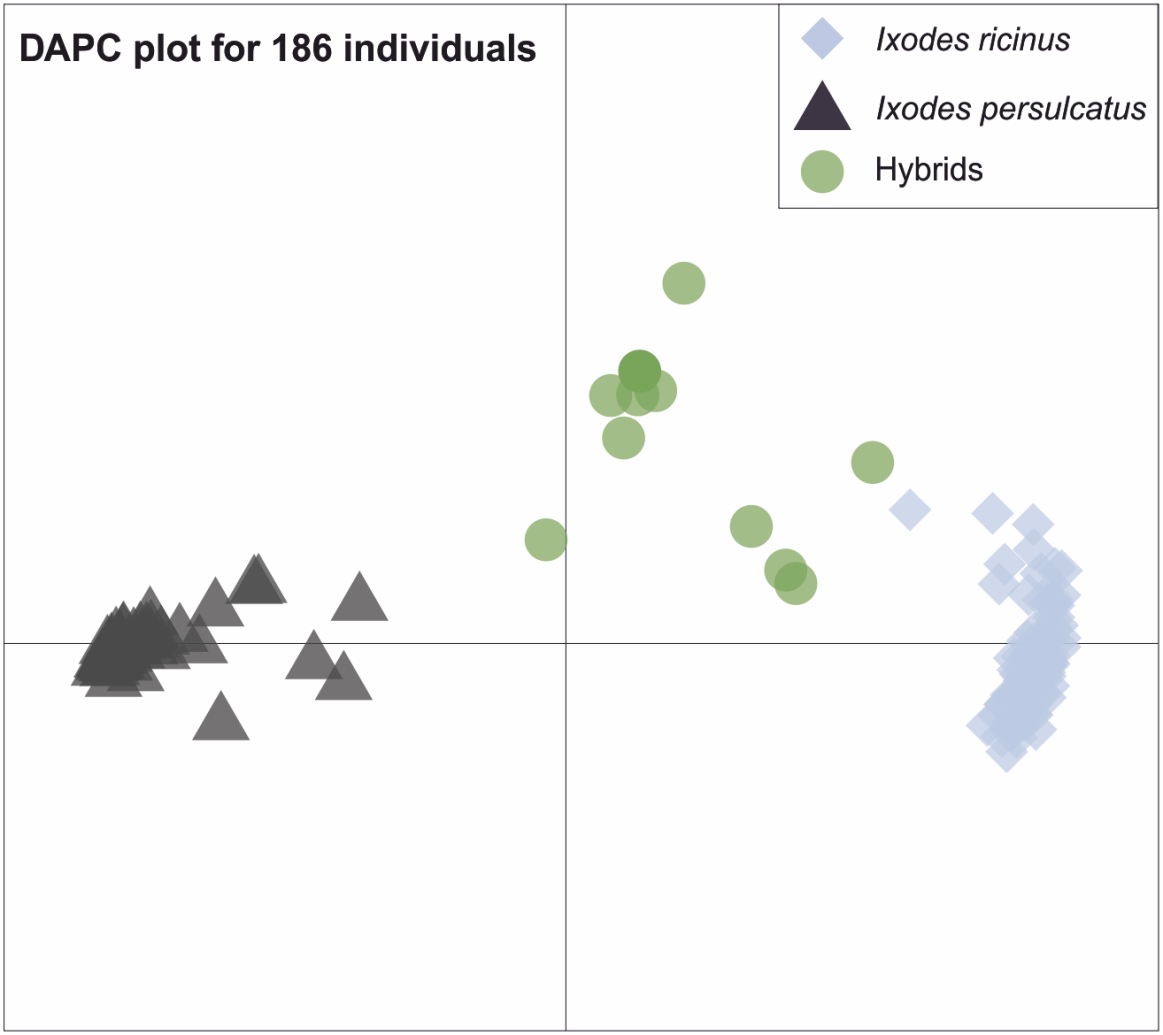
DAPC plot produced by the *R package adegenet*. Coloured shapes black triangles = *Ixodes ricinus* cluster; blue diamonds = *Ixodes persulcatus* cluster; green circles = putative hybrids cluster). Cluster sizes comprise of 88, 87 and 11 for the three clusters respectively.

Only one principal component was retained for the DAPC‐based a‐score optimization. Overall, the DAPC analysis identified three distinct clusters (Figure 3). Each cluster corresponded to a posterior group size of 88 (cluster 1, *I. ricinus*), 87 (cluster 2, *I. persulcatus*) and 11 (cluster 3, putative hybrids). Structure analysis inferred possible genetic clusters (i.e., *K* = 2) (see supplementary material Figure S4). Further analysis for the optimal *K* clusters using structure harvester (Evanno et al., 2005) identified *K* = 2 (Figure S3 and Table S2). The inferred *K* = 2 clusters corresponded to the pre‐defined species groups based on their membership proportions indicating the possible accurate assignment of the two tick species by structure analysis. Membership proportions for each of the two pre‐defined species were found to be 0.964 and 0.941 for *I. ricinus* and *I. persulcatus* clusters respectively, with admixed individuals showing ancestral proportions of 0.036 and 0.059 for both species.

**FIGURE 4:**
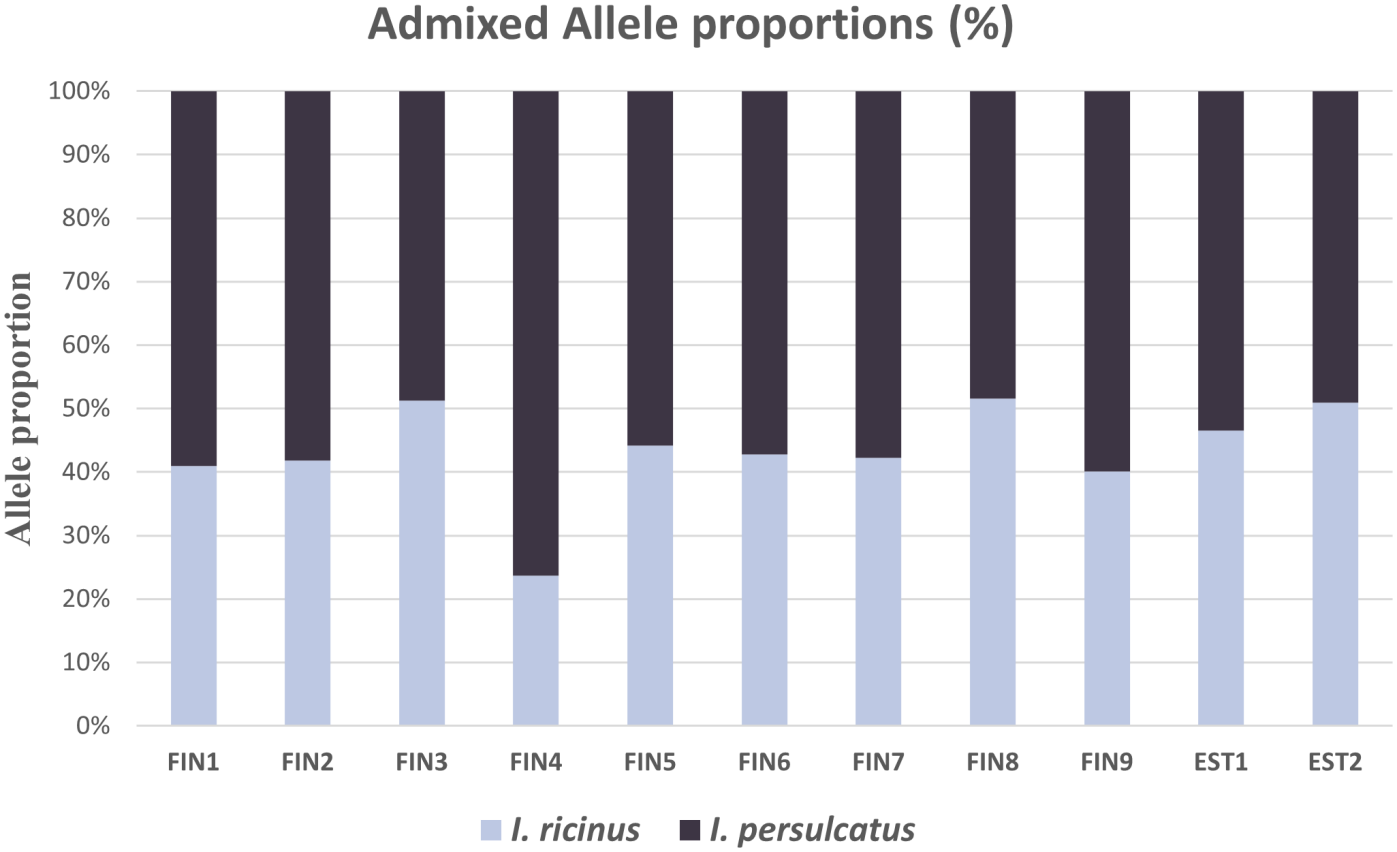
Allele proportions (%) among 11 putative hybrids from Finland (FIN: *n* = 9) and Estonia (EST: *n* = 2).

For hybrid identification, the results from the DAPC scatter plot analysis produced three main clusters (Figure 3). From this, we identified the presence of 11 potential hybrid individuals as a distinct cluster from the two main species clusters. Similarly, allele frequency plots from Structure software analysis identified 11 admixed individuals with significant allele proportions ranging from 24% to 76% for both species (Figure 4). Despite the varying allele proportions, nine of these hybrids were morphologically identified as *I. ricinus*, and only two were *I. persulcatus*. The result indicates possible hybridization between the two species within locations where species occurrence likely overlaps. Two of the potential hybrids were found among the Estonian samples, while the remaining nine came from the Finnish crowdsourced samples. Allele frequencies of four of the detected putative hybrid individuals were near the Mendelian 50:50 proportions (Figure 4).

### 3.3 Populations genetic diversity indices

Computed diversity estimates of the two species revealed that *I. ricinus* had on average a higher number of private alleles compared to *I. persulcatus* (Table 1), but the same number of variant sites for both species. Moreover, average observed heterozygosity indices differed between *I. ricinus* and *I. persulcatus* (Table 1). For example, *I. persulcatus* populations had on average lower *F*_ST_ estimate (*F*_ST_ = 0.02 ± 0.18, see supplementary material A, Table S3) compared to *I. ricinus* populations (*F*_ST_ = 0.04 ± 0.17, see supplementary material A, Table S4). Nucleotide distances between the two species were found to be 0.52, while average expected heterozygosity between individuals in the same cluster were found to be 0.28 and 0.12 for *I. ricinus and I. persulcatus* clusters respectively.

**TABLE 1:** Pure species diversity indices. The table consist of only 175 pure species diversity statistics, IxoPERS (*Ixodes persulcatus*), IxoRICI (*Ixodes ricinus*), number of private alleles (P_alleles), number of samples per locus (Num_individuals), allele frequency (P), observed heterozygosity (Obs_Het) and expected heterozygosity (Exp_Het).

Pop_ID	P_alleles	Num_individuals	*p*	Obs_Het	Exp_Het
Ixo_PERS	643	83.3 ± 0.05	.9	0.05	0.08
Ixo_RICI	1899	77.5 ± 0.08	.9	0.09	0.16

The results from our structure analysis reveal similar patterns between the same species population as revealed by the *F*_ST_ estimates. The results from both DAPC and STRUCTURE clustering methods identified a strong genetic structure between *I. ricinus* and *I. persulcatus* (Figure 3 and Figure S4). We also identified structuring for both species when only pure species were analysed without putative hybrids (Figures S5–S7). Interestingly, we observed structuring within the *I. ricinus* cluster when hybrids were removed and only pure species were analysed, but the same was not true for *I. persulcatus* (Figures S8 and S9). These results indicate that (a) the presence of hybrids may interfere with analyses of within species structure and (b) the species *I. ricinus* is apparently more structured.

## 4 DISCUSSION

In this study, we investigated the ability of RAD‐generated SNPs to identify the two common tick species, *I. persulcatus* and *I. ricinus*, and their hybrids. We further examined the levels of differentiation between and within species. Using two different clustering approaches, our results demonstrate the ability of RADseq to assign the two tick species and identify hybrids between them.

Our SNPs were able to discriminate between the two species and identify potential hybrids. Both clustering methods concordantly identified the two tick species and respective hybrids. Out of the 186 individuals tested, 11 hybrids were identified. We detected both male and female hybrids in a ratio of 1:10 from all samples, highlighting the utility of the RAD makers for hybridization studies. Allele frequencies of four of the detected hybrid individuals were near Kovalev and colleagues' description of hybrids based on the ITS2 region (Figure 3). These allele proportions suggest that most of the identified admixed individuals are first generation hybrids (offspring of pure *I. ricinus* and pure *I. persulcatus*). The fourth individual identified as a hybrid from the Finnish samples (FIN4) had almost 24%:76% allele proportions for *I. ricinus*: *I. persulcatus* respectively, suggesting the possibility of backcrossing, or mating of viable hybrid individuals with the parental species. While further investigation regarding the timing of hybridization events is necessary, these results are similar to Litov et al. (2022) on the differentiation of laboratory generated hybrids between *I. ricinus* and *I. persulcatus*. Although evidence is still lacking for ticks, an example from the malaria vector *Anopheles gambiae* demonstrates that the emergence of novel genotypes and eventual phenotypes by hybridization could serve as the basis for geographical range expansion and the origin of novel taxa of pathogens and vectors (Arnold, 2004). Hybridization could possibly generate relatively more aggressive populations with wider host range compared to parental species populations (Uspensky, 1993). Apart from the potential evolutionary and epidemiological consequences of hybridizations between distant disease vectors, several other reports exist that recombination between divergent microorganisms may give rise to novel human pathogens (Cogliati et al., 2001; Gibbs et al., 2001; Kroll et al., 1998; Laird et al., 2003; Machado & Ayala, 2001; Smoot et al., 2002; Webster, 2001). Hybridization among tick species has the potential to bring divergent microorganisms together. Evidence abounds for the need to identify hybrids in tick populations in Finland especially when this involves the two most important disease vectors responsible for the spread of Lyme disease and TBEV in Eurasia.

Comparing the average observed heterozygosity estimates between the two species, we found that *I. ricinus* has a higher observed heterozygosity than *I. persulcatus*, as well as a higher number of private alleles. When comparing the native *I. ricinus* with the non‐native *I. persulcatus*, we would expect lower diversity among non‐native populations because of potential genetic bottlenecks after founder effects (Allendorf & Lundquist, 2003). Indeed, the low genetic diversity levels as well as lack of genetically differentiated populations in *I. persulcatus* could be due its recent introduction into Finland (Sormunen et al., 2023). However, we could not investigate the timing and frequency of introductory events and certainly a larger data set would be required to resolve this.

For a native species it is expected that *I. ricinus* would possess relatively higher genetic diversity than its non‐native counterpart as a function of time since establishment. Similarly, the detection of population structure within *I. ricinus* can be justified due to multiple factors that restrict gene flow or promote isolation between different groups within the population (González‐Castellano et al., 2020).

## 5 CONCLUSION

Our study utilized RAD‐generated SNPs to effectively distinguish between the two common tick species, *I. persulcatus* and *I. ricinus*, as well as identify hybrids. The application of two clustering methods demonstrated the robustness of RADseq in species and hybrid identification. Out of the 186 individuals tested, our results identified 11 hybrids, with allele frequency analysis suggesting the presence of first‐generation hybrids and indicating the possibility of backcrossing in one case. To better understand the role and impact of hybridization on these two vectors and the associated pathogens they carry will require further studies. From a population genetic perspective, more representative sample sizes and broader geographic sampling range would be required also to identify hybrid zones and resolve intra‐specific population structure. It is also important to note that the quality of DNA for tick RADseq should be taken into consideration as this could influence the final results. Nevertheless, we argue that our study serves as the basis for future studies in areas related to the likelihood and impact of hybridization as a function of the introduction (via range expansion) of non‐native species.

## AUTHOR CONTRIBUTIONS

**Theophilus Yaw Alale:** Conceptualization (lead); formal analysis (lead); methodology (equal); project administration (equal); validation (equal); visualization (equal); writing – original draft (lead); writing – review and editing (lead). **Jani J. Sormunen:** Conceptualization (equal); data curation (equal); funding acquisition (equal); project administration (equal); resources (equal); supervision (equal); writing – review and editing (equal). **Eero J. Vesterinen:** Conceptualization (equal); data curation (equal); funding acquisition (equal); methodology (equal); project administration (equal); supervision (equal); writing – review and editing (equal). **Tero Klemola:** Data curation (equal); funding acquisition (equal); project administration (equal); resources (equal); supervision (equal); writing – review and editing (equal). **K. Emily Knott:** Funding acquisition (equal); methodology (equal); project administration (equal); supervision (equal); validation (equal); writing – review and editing (equal). **Miguel Baltazar‐Soares:** Conceptualization (equal); formal analysis (equal); investigation (equal); methodology (equal); supervision (equal); visualization (equal); writing – review and editing (equal).

## Supporting information


Data S1.

## Data Availability

The data that support the findings of this study have been openly deposited at NCBI under the Bioproject ID: PRJNA1021372. The submission can be viewed using the following link: http://ftp‐trace.ncbi.nlm.nih.gov/sra/review/SRP463351_20240402_100901_bd70aeebacaadd02f48f547e9276258e. All the codes and scripts generated for data analysis can be found using the links provided under section C of the supplementary materials.
