# Supplementary material for: Genomic signatures of hybridization between Ixodes ricinus and Ixodes persulcatus in natural populations
Source: Ecol Evol. 2024 May 20;14(5):e11415. doi: 10.1002/ece3.11415 (PMC11103643; doi:10.1002/ece3.11415)
Supplement: Supplementary file 1 — Data S1. [file ECE3-14-e11415-s001.docx]

**Supplementary materials (A)**

Table S1. Table of sample(s) origins showing specified geographical locations provided by mailers, number of individual (s) per mail, identified tick species (*Ixodes persulcatus* and *Ixodes ricinus*), and the host species from which the ticks were collected from. The table consist of locations across Finland providing 172 individual ticks, and 14 samples from Estonia (unknown host). Locations marked with asterisk (*) indicate hybrid locations.

| location | Sample size | Species | Host Species |
| --- | --- | --- | --- |
| Espoo | 5 | *I. ricinus* | Human |
| Hailuoto | 4 | *I. per sulcatus* | Human |
| Hanko | 5 | *I. ricinus* | Human |
| Helsinki | 9 | *I. ricinus* | Human |
| Ii | 6 | *I. ricinus* | Human |
| Ii | 7 | *I. persulcatus* | Dog |
| Kaskinen | 6 | *I. persulcatus* | Cat |
| Kemi | 5 | *I. persulcatus* | Dog |
| Kemi | 8 | *I. ricinus* | Dog |
| Oulu | 5 | *I. persulcatus* | Human |
| Kokkola | 7 | *I. ricinus* | Human |
| Kokkola | 6 | *I. persulcatus* | Human |
| Kuopio | 5 | *I. ricinus* | Cat |
| Helsinki | 5 | *I. persulcatus* | Human |
| Lempäälä | 7 | *I. persulcatus* | Dog |
| Maalahti* | 6 | *I. persulcatus* | Human |
| Martiniemi | 5 | *I. persulcatus* | Human |
| Mutala | 6 | *I. ricinus* | Dog |
| Oulu* | 4 | *I. ricinus* | Dog |
| Outokumpu | 6 | *I. persulcatus* | Dog |
| Vaasa | 6 | *I. ricinus* | Dog |
| Parainen | 7 | *I. ricinus* | Human |
| Parainen | 5 | *I. ricinus* | Cat |
| Parainen | 9 | *I. ricinus* | Dog |
| Siilin | 6 | *I. persulcatus* | Dog |
| Siilin | 6 | *I. ricinus* | Human |
| Simoniemi | 3 | *I. persulcatus* | Dog |
| Tampere* | 4 | *I. persulcatus* | Dog |
| Totti* | 5 | *I. persulcatus* | Human |
| Talli/Estonia | 8/6 | *persulcatus/ricinus* | N/A |
| Ukkola | 4 | *I. persulcatus* | Dogs |

Table S2. Evano table output for delta K values. Highlighted row indicates the true K values (K = 2).


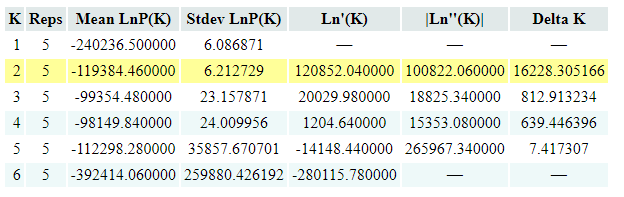


Table S3. Pairwise differentiation (*F*_ST_) estimates between *I. persulcatus* populations

|  | Tampere Hailuoto Ii Kaskinen Kemi Kokkola Simoniemi Lempääla Maalahti Helsinki Outokumpu Totti |
| --- | --- |
| Tampere |  |
| Hailuoto | 0.03834 |
| Ii | 0.00844 0.03887 |
| Kaskinen | 0.01023 0.03257 0.01185 |
| Kemi | 0.01185 0.03573 0.00992 0.01075 |
| Kokkola | 0.02222 0.02846 0.02484 0.02344 0.02094 |
| Simonimi | 0.01921 0.01835 0.02326 0.01893 0.01605 0.00156 |
| Lempäälä | 0.02544 0.00552 0.01969 0.01766 0.02012 0.02456 0.01211 |
| Maalahti | 0.05920 0.03327 0.06019 0.05296 0.04182 0.04943 0.03632 0.03452 |
| Helsinki | 0.04181 0.01409 0.03810 0.03757 0.02847 0.03281 0.01970 0.01181 0.02206 |
| Outokumpu | 0.00852 0.01919 0.01406 0.01386 0.01259 0.01895 0.01050 0.01228 0.04360 0.02686 |
| Totti | 0.02453 0.05608 0.02510 0.02651 0.02526 0.03247 0.03028 0.03482 0.07443 0.05883 0.02667 |

Table S4. Pairwise differentiation (*F*_ST_) estimates between *I. ricinus* populations

|  | Kokkola Parainen Tampere Helsinki Espoo Hanko Kuopio Kemi Siilin Totti Mutala Vaasa Oulu |
| --- | --- |
| Kokkola |  |
| Parainen | 0.00354 |
| Tampere | 0.02599 0.02352 |
| Helsinki | 0.00679 -0.00463 0.02100 |
| Espoo | 0.01348 0.01675 -0.01852 0.01975 |
| Hanko | 0.00483 0.00850 0.01167 0.00176 0.01013 |
| Kuopio | 0.00685 0.00385 0.02752 0.00184 0.02342 0.00654 |
| Kemi | 0.00435 0.00743 0.02647 0.00740 0.01405 0.01013 0.01652 |
| Siilin | -0.00474 0.00256 0.01980 0.00794 0.02132 0.00236 0.01978 0.00919 |
| Totti | 0.03464 0.06034 0.03404 0.02763 0.02274 0.03041 0.04603 0.07143 0.02051 |
| Mutala | 0.01297 0.01462 0.00553 0.00193 0.00734 0.00330 0.02085 0.02032 0.00768 0.11980 |
| Vaasa | 0.01211 0.00576 0.01370 0.00068 0.01259 0.01051 0.00391 0.00672 0.01823 0.06910 0.00937 |
| Oulu | 0.01339 0.01046 0.01574 0.00137 0.02500 0.00476 0.01741 0.00885 0.01455 0.04637 0.01343 0.01719 |


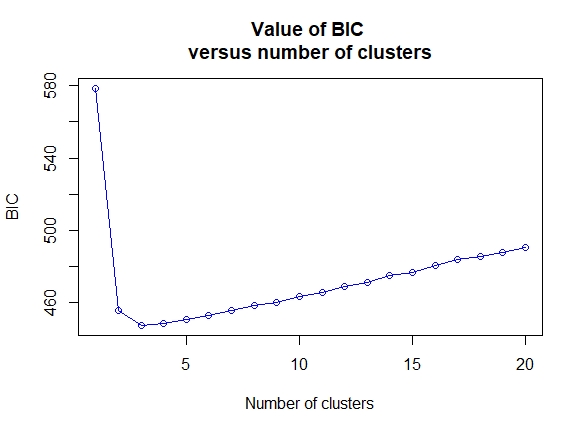


Fig S1. BIC values against the number of clusters. Three clusters were retained.


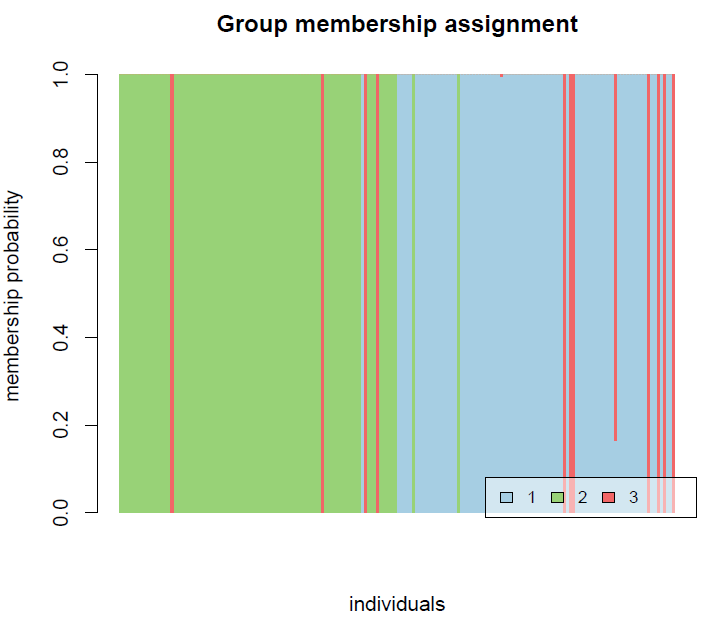


Fig. S2. Group membership assignment based on membership probabilities. Putative hybrids are shown in cluster 3 (red bars).


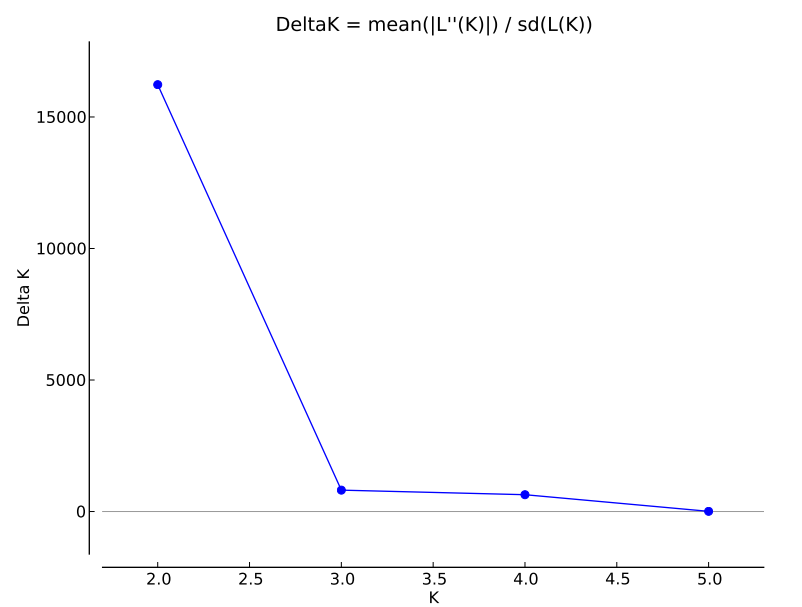


Fig: S3. Optimal *K* value for Delta *K* = 2.


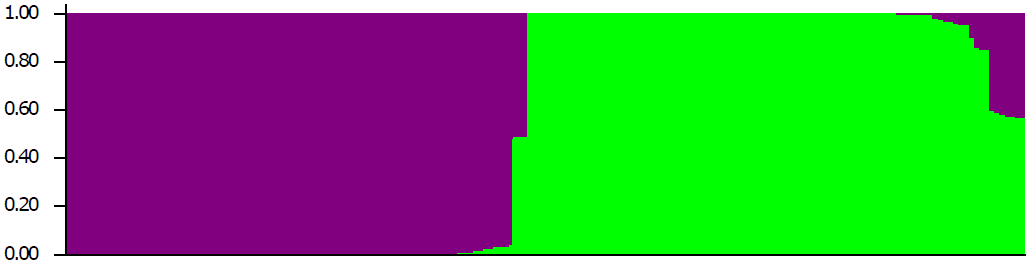


K= 2


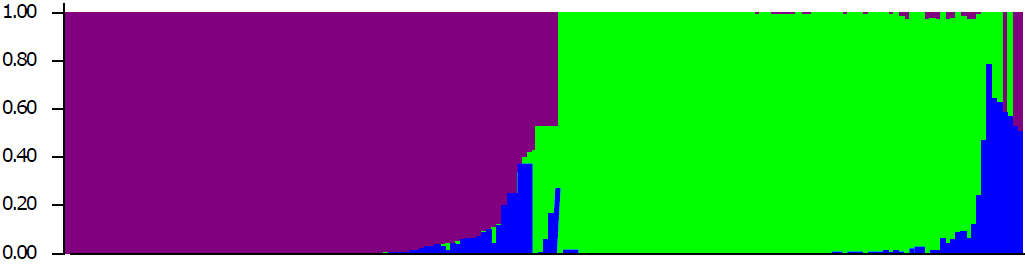


K = 3


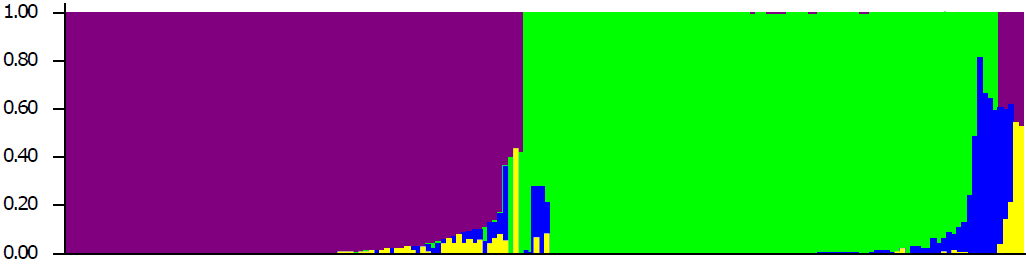


K= 4


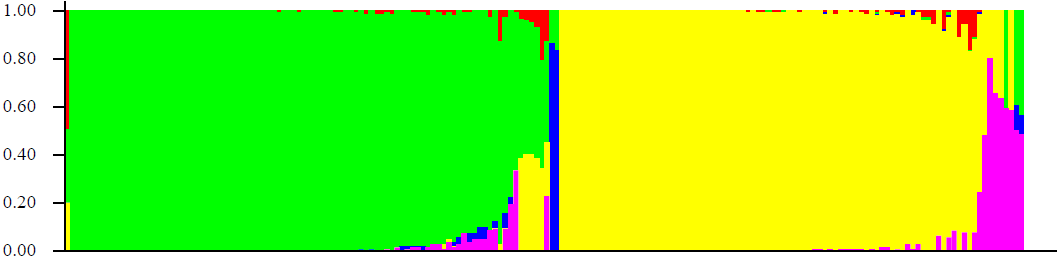


K= 5

Fig S4. Original structure plots for all samples from both species (n = 186). Evano’s number of clusters (*K* = 2).


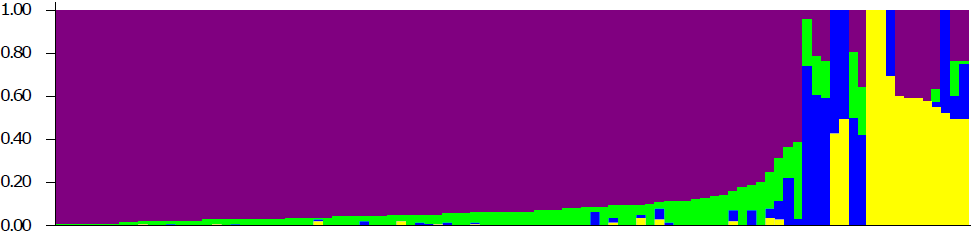


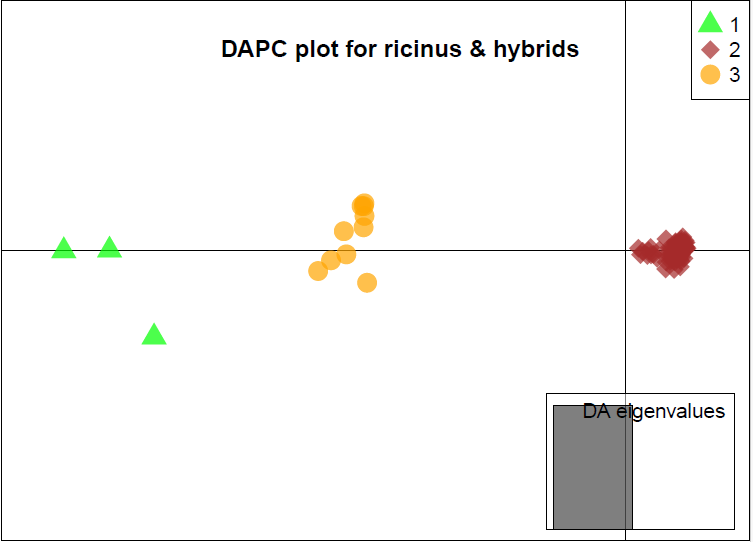


Fig. S5 (a) Structure bar plot for only *I. ricinus* + hybrids samples (n= 99). *K* = 4 clusters based on Evano’s method in the presence of hybrids. Purple bars indicate *I. ricinus;* yellow bars show hybrids; (b) DAPC clustering. Clusters 1 & 3 indicate putative hybrids while cluster 3 show pure *I. ricinus* species.


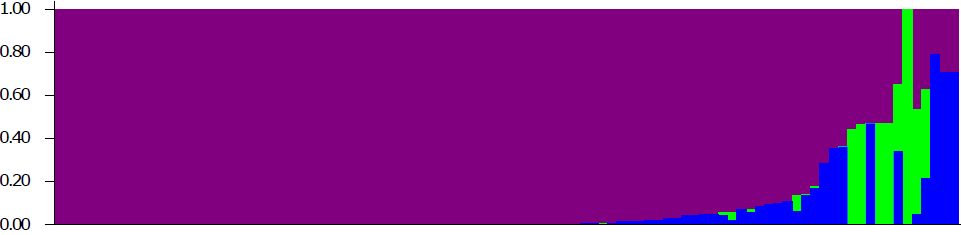


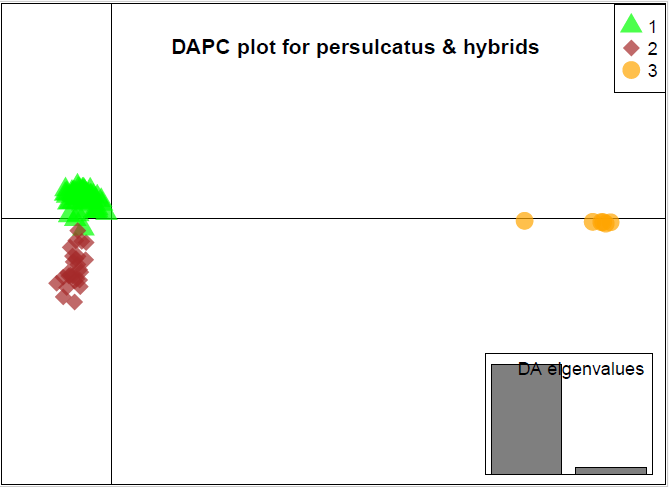


Fig. S6. (a) Structure bar plot for only *I. persulcatus* (purple bars) + hybrids (green bars) (n = 98). *K* = 3 clusters based on Evano’s method in the presence of hybrids. (b) clusters based on DAPC: 1 & 2 indicate *I. persulcatus*; cluster 3 indicates putative hybrids.


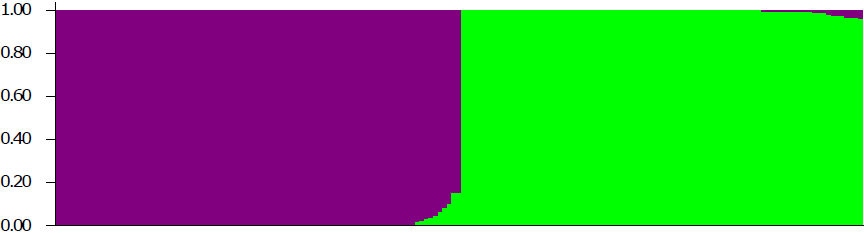


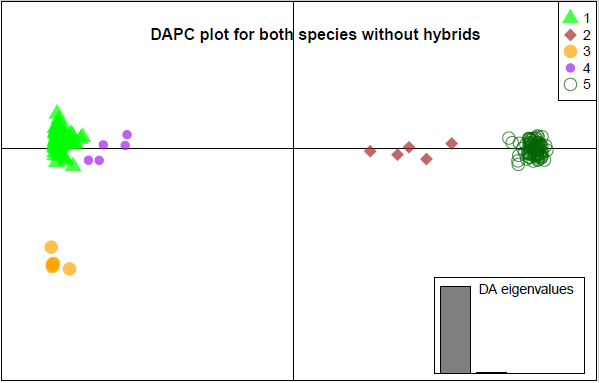


Fig. S7. (a)Original structure plot (*K* = 2) for all samples without putative hybrids (n = 175). Purple colored bars represent *I. ricinus* samples while green colored bars represent *I. persulcatus* individuals; (b) DAPC plot all samples (n = 175; clusters 1, 3 & 4 = *I. Ricinus*; clusters 2 & 5 = *I. persulcatus*)


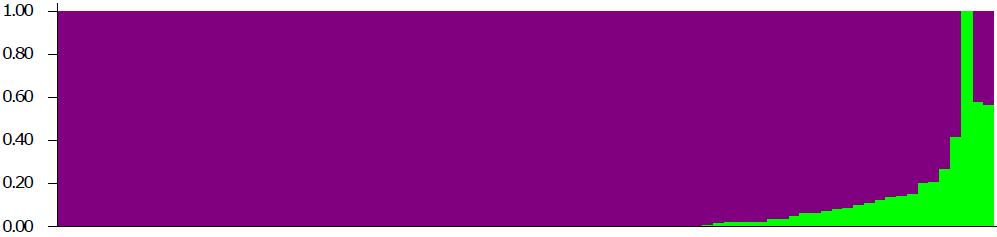


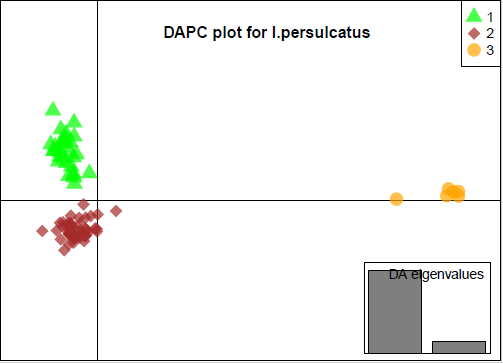


Fig. S8. (a) Structure plot for only *I. persulcatus* (purple bars) group after putative hybrids removed; (b) DAPC plot for *I. persulcatus* individuals (n = 87).


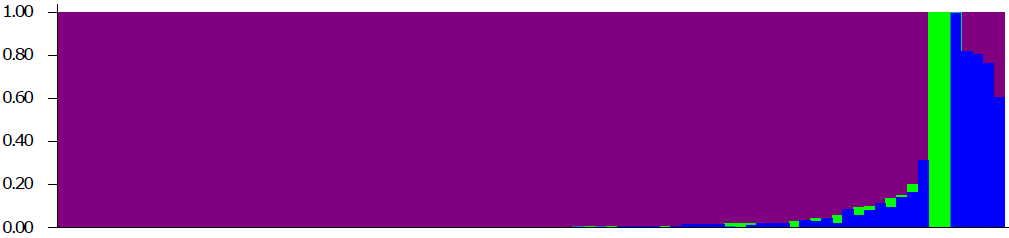


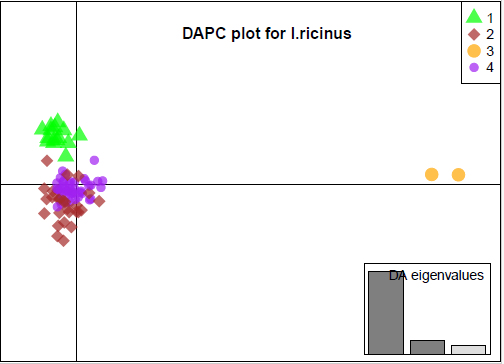


Fig. S9. Structure plot for only *I. ricinus* (purple bars) after putative hybrids removed; (b) DAPC plot for *I. ricinus* individuals (n = 88).

**Supplementary materials (B)**

Comparative analysis using *I. scapularis* reference genome.


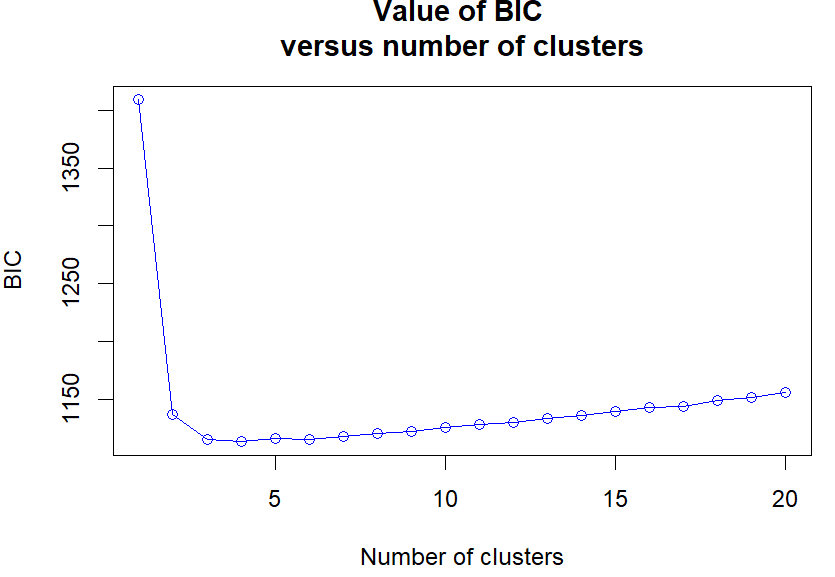


1. Number of clusters suggested for dapc for all samples (n = 186).


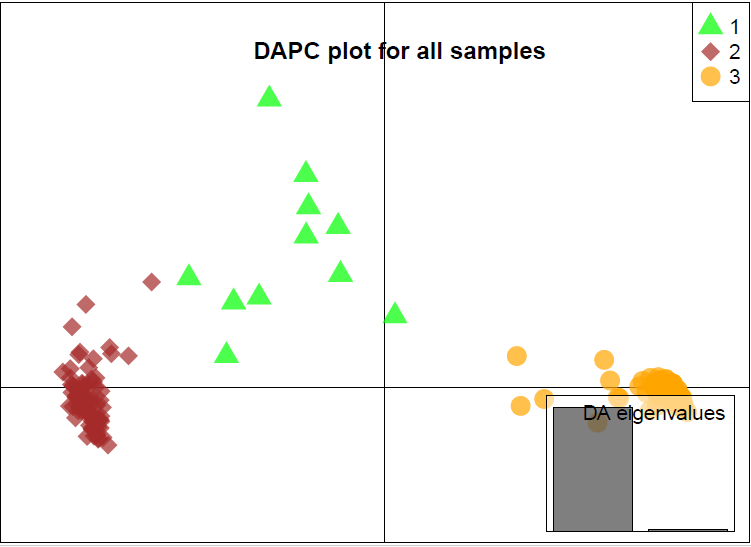


1. DAPC plot for all samples (n = 186). Cluster1 (green color = hybrids); cluster 2 (brown color = *I. ricinus* ); cluster 3 (yellow color = *I. persulcatus*).


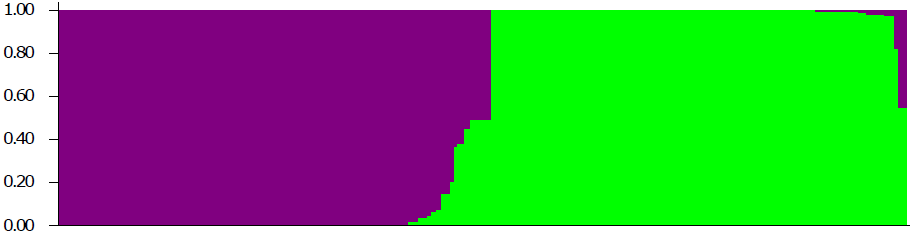


1. Structure plots for all samples for both species (*I. ricinus* =purple; *I. persulcaus* = green; n = 186). Half bars indicate admixed individuals.


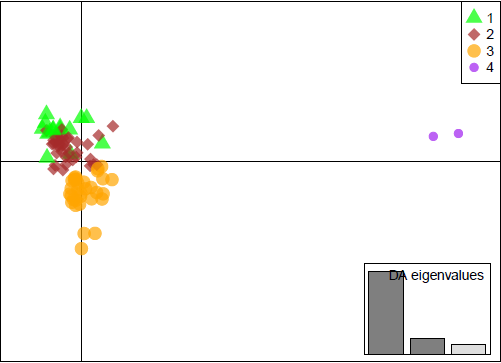


1. DAPC plot for only *I. ricinus* samples (n = 88). No putative hybrid sample included.


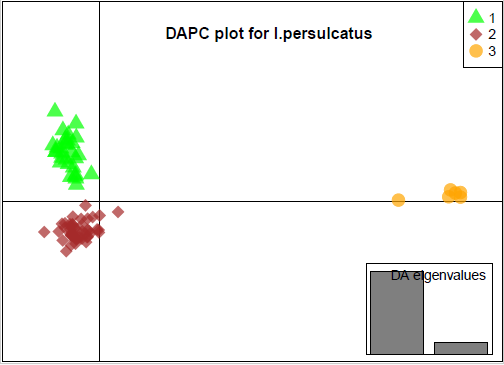


1. DAPC plot for all *I. persulcatus* samples without putative hybrids (n = 87)

**Section C**

Sequence processing, reference genome indexing, alignment, loci catalogue and SNP calling are based on STACKS pipeline (<http://catchenlab.life.illinois.edu/stacks/manual/>). Our shell scripts can be found at the GitHub repository: <https://github.com/theoalal/Shell_scripts>.

The script for DAPC analysis and plots can be found via this link: <https://github.com/theoalal/Bioinformatics_23/blob/main/Tick_dapc_script.R>.
